# Surgical Treatment for Recurrent Refractory Skenitis

**DOI:** 10.1100/tsw.2008.92

**Published:** 2008-07-13

**Authors:** E. P. Miranda, D. C. Almeida, G. P. Ribeiro, J. M. Parente, A. G. Scafuri

**Affiliations:** ^1^ Faculty of Medicine, Federal University of Ceará, Fortaleza, Brazil; ^2^ Faculty of Medicine, Federal University of Vale do São Francisco, Petrolina, Brazil

**Keywords:** skenitis, gynecologic infection, female prostate, sexually transmitted diseases

## Abstract

We report a case of persistent skenitis that was initially misdiagnosed and treated as a urinary infection. Despite an adequate course of antibiotics, the symptoms failed to improve. The case was ultimately resolved with surgical intervention, which resulted in its prompt and complete resolution.

## INTRODUCTION

The Skene's glands, also known as the lesser vestibular, periurethral glands, or paraurethral glands, are structures located on the upper wall of the vagina, around the lower end of the urethra and are normally neither seen nor felt, except in the presence of disease or infection. Close inspection will reveal the pinpoint openings of these periurethral glands. These glands are homologous with the prostate gland in males and seem to be responsible for female ejaculation[1].

## CASE REPORT

A 49-year-old woman presented to our office complaining of dysuria over the past 4 weeks. She had an episode of diverticulitis several months previously, treated with oral antibiotics. Before presenting to us, she had gone to her gynecologist, who diagnosed lower urinary tract infection and prescribed ciprofloxacin 1 g/day. She obtained relief of her symptoms for a couple of days, but they recurred and persisted, despite another course of antibiotics.

She was sexually active with one partner and denied having any previous sexually transmitted diseases. Sexual intercourse was sometimes uncomfortable, but rarely painful. Her menses were somewhat irregular, but she stated that they had always been like that and that it “runs in her family”. She denied having fever, weight loss, vaginal discharge, diarrhea, or constipation.

On physical examination, the patient had normal vital signs and was a moderately overweight woman who appeared well and in no apparent distress. The abdomen was nondistended and nontender to palpation. No inguinal lymphadenopathy was present. On pelvic inspection, she had no vaginal discharge and no cervical motion tenderness was observed on digital exam. The rest of the physical examination was unremarkable.

The urine pregnancy test was negative. Findings on wet-mount examination and urinalysis were normal. Cultures for *Neisseria gonorrhoeae*, *Chlamydia* sp., and unspecific organisms were negative. The patient was referred for pelvic ultrasonography, but the findings were unremarkable to her complaints. Another culture was performed, which was also negative.

A bidigital examination was pursued and a 1- × 1-cm nodule was palpated in the left vaginal wall, close to the opening of the urethra, more specifically at the topography of the left Skene's gland. A purulent discharge was noted at local compression and the diagnosis of skenitis was established (Fig. 1)

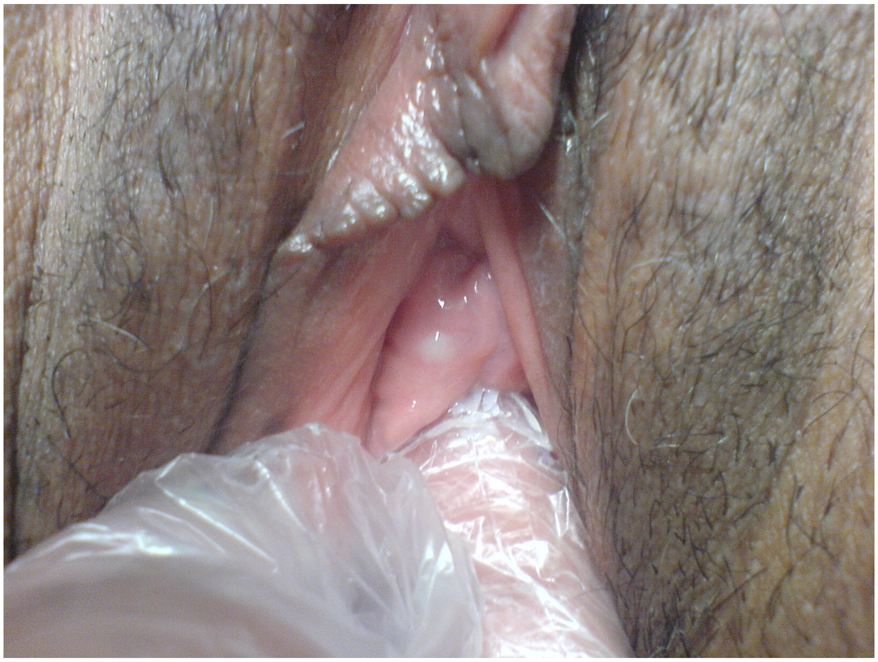

**FIGURE 1**. Local compression at the left vaginal wall, showing purulent discharge at the topography of the left Skene's gland opening.

Surgical treatment was scheduled and successfully performed. It consisted of a simple incision at the opening of the gland to allow drainage of the secretion. There was no history of recurrence and the patient related complete relief of her symptoms.

## DISCUSSION

The Skene's glands have a highly variable anatomy and in some extreme cases, appear to be entirely absent. When infected, the Skene's gland will become enlarged and tender, a condition known as skenitis. Repeated infections may lead to increasing obstruction of the gland and result in a suburethral cyst or an abscess cavity. Eventually, the cavity ruptures into the urethral lumen, creating a communication between the urethral lumen and the suburethral cyst. Repeated pooling of urine into the suburethral cyst during urination may lead to the formation of a urethral diverticulum[1].

The palpation of the Skene's glands can be done by rotating the internal fingers upward and palpating the labia bilaterally; by applying pressure with the index finger on the anterior vaginal wall, a discharge can be obtained[2,3].

In acute gonorrhea, these glands are almost always infected. Cultures, particularly for gonorrhea, should thus be obtained. Some reports showed that α1 blockers and effective antibiotics were considered an ideal choice of treatment for female skenitis. Good options for antibiotics would include those most helpful for treating urethritis: cefixime 400 mg orally in a single dose, ceftriaxone 125 mg i.m. in a single dose, ciprofloxacin 500 mg orally in a single dose, or ofloxacin 400 mg orally in a single dose combined with azithromycin 1 g orally in a single dose or doxycycline 100 mg orally twice a day for 7 days[4].

The surgical cases can be resolved with a simple incision and drainage of the gland, which generally result in complete resolution. Topical anesthetic (20% benzocaine, or "Hurricaine") can be applied to the cyst with a cotton-tipped applicator and allowed to sit for 3–4 min. A single stab wound by a scalpel opens the abscess and allows the drainage of the pus[5].

There may be involvement of these glands in vaginal or vulvar masses. Periurethral lesions occur in almost one-quarter of women with genital masses. The differential diagnosis of an anterior vaginal wall mass should include urethral diverticulum, Skene gland abscess, ectopic ureterocele, Gartner duct cyst, müllerian remnant cyst, and vaginal inclusion cyst. Symptoms vary in women with periurethral masses: pain, dyspareunia, dysuria, and other urinary symptoms are the most common, and will resolve after successful excision of the lesion[4,6].

The reduced number of reported cases of this entity, with only a few descriptions in the literature, led us to believe that this disease is actually underestimated. As the clinical aspects of skenitis are highly variable and are not specific of this condition, it is possible that many misdiagnosed cases are treated as urinary infection and as they obtain complete resolution, surgical treatment is rarely necessary.

## ACKNOWLEDGMENTS

We hereby acknowledge that all authors have significantly contributed to the elaboration of this work, either to the conduction of the case or to the preparation of this manuscript. Informed consent was obtained from the patient and her identity remains unrevealed. No conflicts of interest are attached to this paper.
